# Use of Peak Glucose Level and Peak Glycemic Gap in Mortality Risk Stratification in Critically Ill Patients with Sepsis and Prior Diabetes Mellitus of Different Body Mass Indexes

**DOI:** 10.3390/nu15183973

**Published:** 2023-09-14

**Authors:** Yi-Hsuan Tsai, Kai-Yin Hung, Wen-Feng Fang

**Affiliations:** 1 Division of Pulmonary and Critical Care Medicine, Department of Internal Medicine, Kaohsiung Chang Gung Memorial Hospital, Chang Gung University College of Medicine, Kaohsiung 83301, Taiwan; flyninesun@gmail.com (Y.-H.T.); redrosahung@yahoo.com.tw (K.-Y.H.); 2 Department of Nutritional Therapy, Kaohsiung Chang Gung Memorial Hospital, Kaohsiung 83301, Taiwan; 3 Department of Nursing, Mei Ho University, Pingtung 91202, Taiwan; 4 Department of Respiratory Therapy, Kaohsiung Chang Gung Memorial Hospital, Chang Gung University College of Medicine, Kaohsiung 83301, Taiwan; 5 Department of Respiratory Care, Chang Gung University of Science and Technology, Chiayi 61363, Taiwan

**Keywords:** diabetes, sepsis, peak glucose level, peak glycemic gap, mortality

## Abstract

Sepsis remains a critical concern in healthcare, and its management is complicated when patients have pre-existing diabetes and varying body mass indexes (BMIs). This retrospective multicenter observational study, encompassing data from 15,884 sepsis patients admitted between 2012 and 2017, investigates the relationship between peak glucose levels and peak glycemic gap in the first 3 days of ICU admission, and their impact on mortality. The study reveals that maintaining peak glucose levels between 141–220 mg/dL is associated with improved survival rates in sepsis patients with diabetes. Conversely, peak glycemic gaps exceeding 146 mg/dL are linked to poorer survival outcomes. Patients with peak glycemic gaps below −73 mg/dL also experience inferior survival rates. In terms of predicting mortality, modified Sequential Organ Failure Assessment–Peak Glycemic Gap (mSOFA-pgg) scores outperform traditional SOFA scores by 6.8% for 90-day mortality in overweight patients. Similarly, the modified SOFA-Peak Glucose (mSOFA-pg) score demonstrates a 17.2% improvement over the SOFA score for predicting 28-day mortality in underweight patients. Importantly, both mSOFA-pg and mSOFA-pgg scores exhibit superior predictive power compared to traditional SOFA scores for patients at high nutritional risk. These findings underscore the importance of glycemic control in sepsis management and highlight the potential utility of the mSOFA-pg and mSOFA-pgg scores in predicting mortality risk, especially in patients with diabetes and varying nutritional statuses.

## 1. Background

Sepsis is a condition characterized by a dysregulated host response to infection, often leading to high mortality rates [1]. Dysglycemia, which involves disordered glucose metabolism, has been associated with poor outcomes in sepsis patients [2]. However, the standard assessment of sepsis severity, the sequential organ failure assessment (SOFA) score [3], does not include measurements of glucose levels. Incorporating blood glucose levels into SOFA scores may enhance patient stratification and guide treatment decisions more effectively.

The prevalence of diabetes as a comorbidity in sepsis patients has been on the rise [4]. The influence of preexisting diabetes on sepsis outcomes remains controversial [5,6,7], particularly when the severity of diabetes is not considered [8,9]. While some studies have reported no significant association between diabetes and sepsis outcomes or mortality [6,7], others have suggested that diabetes could increase the risk of sepsis due to impaired immune responses [10] and disruptions in adaptive immune functions [11]. Furthermore, aside from its established connection to sepsis mortality [12], glucose assumes a pivotal role in the pentose phosphate pathway, wherein it generates and sustains the requisite levels of nicotinamide adenine dinucleotide phosphate (NADPH). This NADPH is crucial for reducing oxidized glutathione, governing redox signaling, and combating infections [13]. Consequently, the blood glucose level may be indicative of the severity of infection in individuals suffering from sepsis. Prior research has highlighted the potential significance of diabetes status and peak glucose levels within the first 3 days of ICU admission for mortality risk assessment in critically ill sepsis patients. Grouping patients based on peak glucose levels may aid in predicting sepsis progression, host immune responses, and outcomes. A previous study introduced a modified SOFA score that incorporated peak glucose levels, enhancing sepsis mortality prediction irrespective of diabetes status [14]. However, this study was limited by its sample size and did not consider baseline glucose levels. 

Glycated hemoglobin (HbA1c) is a widely accepted marker of long-term glycemic control. Patients with lower HbA1c levels may experience less hyperglycemia during severe illness [15]. Therefore, using peak glucose levels to assess sepsis severity may have limitations for patients with well-controlled glycemia. Glycemic variability has been proposed as a better predictor of outcomes in critically ill patients both with and without diabetes [2,12,16,17,18,19]. Increased glycemic variability has been linked to higher mortality rates, an increased need for renal replacement therapy, ventilation use, and shock risk [17], and can therefore be used to predict the mortality of patients with sepsis [12], diabetes [16], and liver abscess [20]. Previous studies have analyzed the glycemic gap using absolute glucose change values instead of measuring glucose level increases or decreases. Some studies have suggested that in the presence of diabetes, hyperglycemia may not necessarily lead to worse outcomes. For example, a study on diabetes patients hospitalized for cardiac disease found U-shaped curves for admission glucose levels, indicating increased mortality associated with both elevated and decreased glucose levels [21]. However, the effects of positive and negative changes in glucose levels compared to chronic glycemia in patients with preexisting diabetes remain unclear.

This study hypothesized that peak glucose levels and peak glycemic gaps within the first 3 days of ICU admission are associated with mortality in critically ill sepsis patients with preexisting diabetes. We introduced novel modified SOFA scores, namely the modified SOFA-peak glucose level (mSOFA-pg) score and the modified SOFA-peak glycemic gap (mSOFA-pgg) score, to aid in mortality risk stratification. To account for different nutritional statuses, we considered the modified Nutrition Risk in the Critically Ill (mNUTRIC) score [22] and body mass index (BMI) for subgroup analysis.

## 2. Methods 

### 2.1. Subjects and Study Design

This retrospective, observational, multicenter cohort study was conducted using the Chang Gung Research Database (CGRD). We extracted information regarding patients with pre-existing diabetes and sepsis who were admitted to various adult intensive care units (ICUs), encompassing both medical and surgical units, for their inaugural ICU stay during the period spanning 2012 to 2017. Data were obtained from the electronic records of six branches within the Chang Gung Memorial Hospital network, specifically, two tertiary referral hospitals (Linkou and Kaohsiung) and four regional hospitals (Taipei, Chiayi, Taoyuan, and Keelung), boasting a collective bed capacity of approximately 9000 beds. The CGRD dataset was meticulously curated with rigorous quality assurance procedures overseen by the Chang Gung Medical Foundation. It is closely integrated with Taiwan’s National Health Insurance reimbursement system, ensuring the utmost accuracy and comprehensiveness of the data. However, it is important to acknowledge certain potential limitations within the database, primarily stemming from missing values that may not have been recorded in electronic medical records. Previous research has demonstrated that CGRD surpasses the National Health Insurance Research Database [23] in terms of its depth of clinical information, encompassing essential elements such as pathological and laboratory results. Notably, this database offers precise temporal sequencing and laboratory data, meticulously aligned with individuals’ electronic health records. This study was approved by the Institutional Review Board (No. 201801213B0C501, date of approval 8 June 2019) of Chang Gung Medical Foundation.

The participant selection process is illustrated in Figure S1. Peak blood glucose levels were assessed within the first three days following admission to the ICU, irrespective of whether patients received acute glucose-lowering interventions. HbA1c levels measured in the three months preceding ICU admission were obtained from available data. Specifically, we used the data points closest in chronological proximity to the date of ICU admission. Patients were excluded from the analysis if data regarding HbA1c or peak glucose levels were not accessible.

### 2.2. Definitions and Criteria

Diabetes was defined as patients having a prior diagnosis, indicated by the International Statistical Classification of Diseases and Related Health Problems, Ninth Revision (ICD-9) codes 2500, 2504, 2505, 2506, 2507, 2508, or 2509, or corresponding codes in the Tenth Revision (ICD-10), recorded before their admission to the ICU. Screening for sepsis was conducted using ICD-9 or ICD-10 codes. Additionally, patients with infections accompanied by acute organ dysfunction that met the criteria outlined in the Third International Consensus Definitions for Sepsis and Septic Shock (Sepsis-3) [1,24] were also included.

The baseline glucose level was determined by the glucose measurement taken upon admission to the ICU. The peak glucose level was defined as the highest glucose value recorded during the initial three days of ICU admission [14]. Patients were stratified into three groups based on their peak glucose levels (≤140, 141–220, and >220 mg/dL) and into three groups based on their HbA1c levels (<6.5%, 6.5–7.9%, and ≥8.0%; see Table 1). To represent glycemic variability, we calculated the peak glycemic gap by subtracting the estimated average glucose level (28.7 × HbA1c − 46.7) from the peak glucose level [25]. Following previous research, we used cutoff values of 72 mg/dL [20] and 146 mg/dL [18] to categorize patients into the following groups: below −72, −72 to −1, 0 to 72, 73 to 146, and above 146 mg/dL. Furthermore, considering our prior study, we established a cutoff value of 6 for the mNUTRIC score [22] (Table S1). Patients were categorized into three groups based on their BMI: underweight (<18.5 kg/m^2^), normal-weight (18.5–24.9 kg/m^2^), and overweight (≥25.0 kg/m^2^).

Each component of the SOFA score is graded on a scale of 0–4, yielding a total score range of 0–24 points. In this study, two new parameters, peak glucose level and peak glycemic gap, were introduced. A score of 4 was assigned when the peak glucose level fell outside the range of >500 or <80 mg/dL, indicating a significantly elevated risk of in-hospital mortality. Peak glucose levels ranging from 301–500 and 81–110 mg/dL received a score of 3, while levels between 221–300 mg/dL were assigned a score of 2. Glucose levels within the ranges of 111–140 mg/dL and 141–220 mg/dL were, respectively, scored as 1 and 0. The mSOFA-pg scores were calculated as the sum of the SOFA scores on Day 3 and the peak glucose level scores.

Additionally, taking into account the distinction between positive and negative changes in glucose levels, we introduced a new metric, the peak glycemic gap, based on insights from survival curve analyses. A score of 4 was allocated to cases where the peak glycemic gap exceeded 146 mg/dL, indicating elevated mortality risk. For those with a peak glycemic gap below −72 mg/dL, a score of 3 was assigned, while gaps falling within the range of −72 to −1 or 73 to 146 mg/dL received a score of 1. A gap of 0 to 72 was scored as 0. The mSOFA-pgg scores were computed as the sum of the SOFA scores on Day 3 and the peak glycemic gap scores.

### 2.3. Data Collection

Various baseline characteristics and clinical data were collected, including peak blood glucose levels, HbA1c levels, SOFA scores [26,27,28], Charlson comorbidity index (CCI) scores, Diabetes Complications Severity Index (DCSI) scores, hospital outcomes (7-day, 28-day, and 90-day), and in-hospital mortality.

### 2.4. Statistical Methods

Clinical characteristics and outcomes were summarized using frequencies and percentages for categorical variables, and for continuous variables, we presented means ± standard deviations (SDs) or medians, as appropriate. While means ± SDs were the preferred method for expressing the coefficient of variability, we employed the Mann–Whitney U test or Kruskal–Wallis test, as appropriate, for non-normally distributed data. Between-group differences for continuous variables, such as HbA1c categories (<6.5%, 6.5–7.9%, and ≥8.0%), were assessed using the Mann–Whitney U test. Categorical variables were analyzed with a chi-square test. Nonparametric Kruskal–Wallis tests were employed as alternatives to assess variance among groups for non-normally distributed continuous variables, followed by Dunn–Bonferroni pairwise tests for post hoc comparisons.

To compare survival outcomes across groups, we constructed Kaplan–Meier (KM) survival curves and conducted log-rank tests, applying a Sidak correction to *p*-values. Mortality hazard ratios (HRs) between groups were assessed using the Cox proportional hazards model. Univariable and multivariable logistic regression analyses were performed to determine the mortality HRs, considering both models and baseline characteristics. Logistic regression was utilized to evaluate odds ratios of mortality, considering the SOFA, mSOFA-pg, and mSOFA-pgg scores. Model fit was assessed using the Nagelkerke R-squared test. Statistical significance was set at a two-sided *p*-value of <0.05. All statistical analyses were conducted using SAS 9.4 TS Level 1 M2 (X64_8PRO platform; SAS Institute, Cary, NC, USA).

## 3. Results

### 3.1. Baseline Characteristics of Patients with Sepsis, Stratified by HbA1c

This study encompassed a total of 4847, 6050, and 4987 patients diagnosed with sepsis, categorized into three groups based on their HbA1c levels: <6.5%, 6.5–7.9%, and ≥8.0% (see Table 1). Notably, the group with HbA1c ≥ 8.0% was characterized by a younger age profile (mean age = 60.9, *p* < 0.001), the highest mean body mass index (BMI; 24.6 kg/m^2^, *p* < 0.001), and the lowest proportion of females (40.1%, *p* < 0.001). Additionally, this group exhibited the lowest percentages of patients with pneumonia (33.8%, *p* < 0.001), urinary tract infections (17.4%, *p* < 0.001), vasopressor use (21.8%, *p* = 0.048), ventilator use (49.9%, *p* < 0.001), and hemodialysis (17.2%, *p* < 0.001).

Upon admission to the ICU, the HbA1c ≥8.0% group displayed the lowest median APACHE II scores on both Day 1 (15, *p* < 0.001) and Day 3 (13, *p* < 0.001), as well as the lowest average pulse pressure on Day 1 (59 mmHg, *p* < 0.001) and Day 3 (60 mmHg, *p* < 0.001). Moreover, they exhibited the highest average white blood cell counts on Day 1 (11,100, *p* < 0.001) and Day 3 (9300, *p* < 0.001). Conversely, patients with HbA1c < 6.5% had the highest average Charlson Comorbidity Index (CCI) scores (4.0, *p* < 0.001) and SOFA scores on both Day 1 (5.0, *p* = 0.010) and Day 3 (6.0, *p* < 0.001).

**Figure 1:**
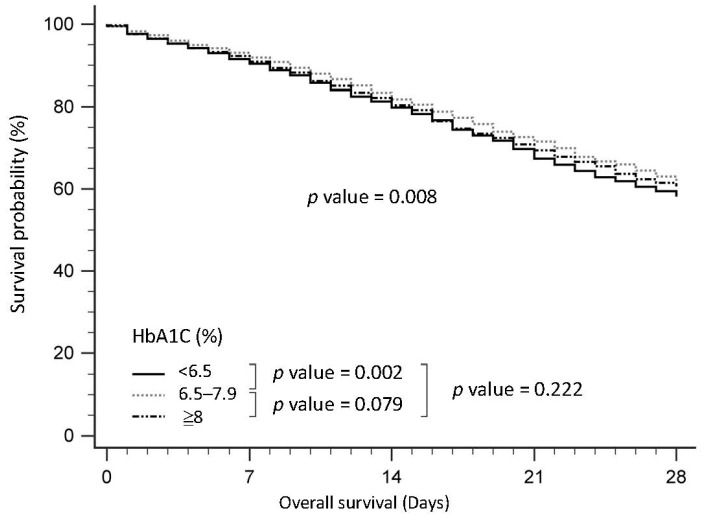
Survival curve for the patients with sepsis in the intensive care unit according to HbA1c.

In terms of mortality, patients with HbA1c levels in the range of 6.5–7.9% exhibited the lowest average 7-day mortality rate (6.4%, *p* = 0.025), while those with HbA1c ≥ 8.0% had the lowest average rates for 28-day (15.7%, *p* < 0.001), 90-day (21.2%, *p* < 0.001), and in-hospital mortality (21.5%, *p* < 0.001). Additionally, we categorized HbA1c into two groups (≤7.0% and >7.0%), as shown in Table S2. Figure 1 illustrates the survival curves of patients stratified by HbA1c levels, revealing that patients with HbA1c levels in the range of 6.5–7.9% had a significantly better average survival rate compared to those with HbA1c < 6.5% (*p* = 0.002).

### 3.2. Baseline Characteristics of Patients with Sepsis, Stratified by Peak Blood Glucose

The data presented in Table 1 highlight notable differences among groups categorized by their peak glucose levels. The group with peak glucose levels exceeding 220 mg/dL exhibited characteristics such as the youngest mean age (61.7), the highest proportion of women (40.3%), and the greatest use of vasopressors (22.1%) and ventilators (52.9%), with all comparisons demonstrating statistical significance (all *p* < 0.001). This group also displayed the highest average Diabetes Complications Severity Index (DCSI) score (4.0, *p* < 0.001), APACHE II scores on Day 1 (16, *p* < 0.001), average white blood cell counts on Day 1 (11,900, *p* < 0.001) and Day 3 (7900, *p* < 0.001), and average body temperature on Day 3 (36.6 °C, *p* < 0.001). Conversely, it had the lowest average systemic blood pressure on both Day 1 (132 mmHg, *p* < 0.001) and Day 3 (143 mmHg, *p* = 0.035), as well as the lowest average pulse pressure on Day 1 (58 mmHg, *p* < 0.001).

In contrast, the group with peak glucose levels ≤ 140 mg/dL exhibited the lowest average BMI (24.2, *p* < 0.001) and the highest incidence of urinary tract infections (16.2%, *p* = 0.019) and hemodialysis (19.1%, *p* = 0.018). The group with peak glucose levels between 141 and 220 mg/dL had the lowest mortality rates, with 7-day mortality at 3.8%, 28-day mortality at 10.3%, 90-day mortality at 19.8%, and in-hospital mortality at 14.4%, all of which were statistically significant (all *p* < 0.001).

Conversely, the group with peak glucose levels > 220 mg/dL experienced the highest 7-day mortality (6.6%, *p* < 0.001), while the group with peak glucose levels ≤ 140 mg/dL had the highest 28-day (16.5%, *p* < 0.001), 90-day (21.5%, *p* < 0.001), and in-hospital mortality (22.1%, *p* < 0.001). Further subgroup analysis of patients with peak glucose levels > 140 mg/dL is presented in Table 2, revealing that the group with peak glucose levels between 181 and 220 mg/dL exhibited the lowest average 7-day (3.5%, *p* < 0.001), 28-day (10.1%, *p* < 0.001), and 90-day (13.9%, *p* < 0.001) mortality rates. Supplementary Table S3 provides a subgroup analysis of patients with peak glucose levels ≤ 140 mg/dL, indicating that the group with peak glucose levels ≤80 mg/dL had the highest mortality rates, including 7-day (15.2%, *p* < 0.001), 28-day (30.5%, *p* < 0.001), 90-day (43.4%, *p* < 0.001), and in-hospital mortality (45.5%, *p* < 0.001).

### 3.3. Survival of Patients with Sepsis, Stratified by Peak Blood Glucose

**Figure 2:**
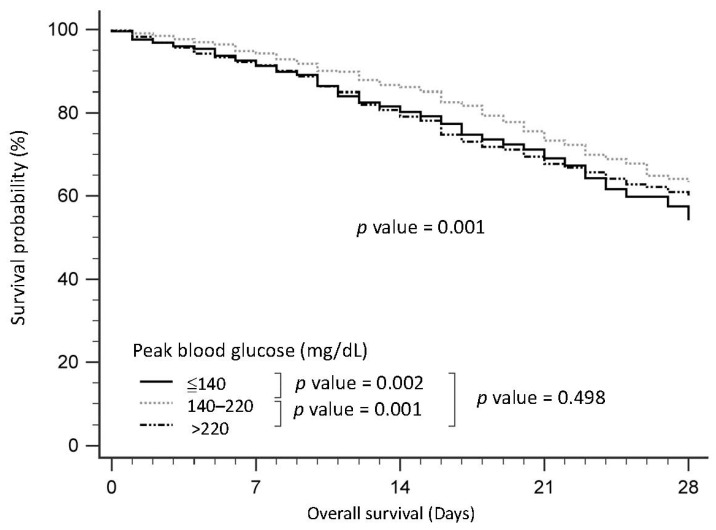
Survival curve for the patients with sepsis in the intensive care unit according to peak blood glucose.

Figure 2 illustrates the survival curves of patients with sepsis, categorized by their peak glucose levels. Notably, patients with peak glucose levels falling within the range of 141–220 mg/dL demonstrated superior survival rates compared to both those with peak glucose levels of <140 mg/dL (*p* = 0.002) and >220 mg/dL (*p* = 0.001).

**Figure 3:**
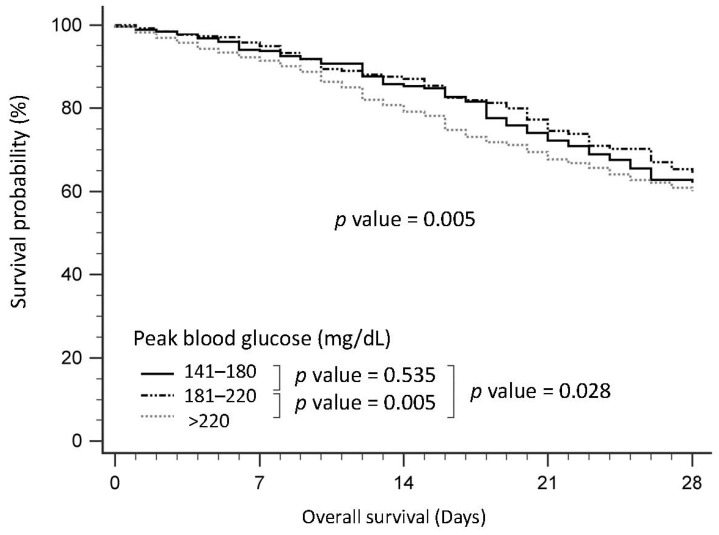
Survival curve of patients (with peak glucose above 140mg/dL) with sepsis in the intensive care unit, according to peak blood glucose.

In Figure 3, we present the survival curves of patients with peak glucose levels exceeding 140 mg/dL. Within this subgroup, those with peak glucose levels surpassing 220 mg/dL exhibited significantly poorer survival outcomes compared to individuals with peak glucose levels between 141–180 mg/dL (*p* = 0.028) or 181–220 mg/dL (*p* = 0.005).

**Figure 4:**
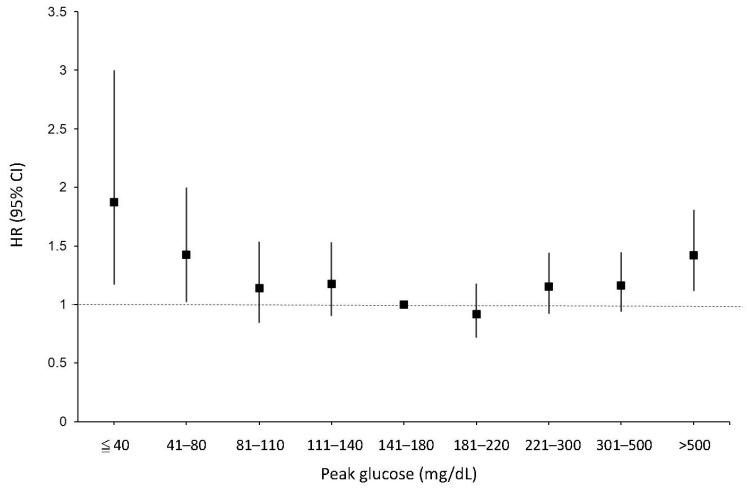
Peak glucose and in-hospital mortality of patients with sepsis in an intensive care unit.

Figure 4 illustrates a U-shaped curve depicting the survival hazard ratios of patients stratified by their peak glucose levels. Interestingly, the nadir of the hazard ratio was observed in patients with peak glucose levels ranging from 181–220 mg/dL. However, it is worth noting that this U-shaped pattern was not evident in the survival hazard ratio of patients stratified by HbA1c, as shown in Figure S2.

### 3.4. Survival of Patients with Sepsis, Stratified by the Peak Glycemic Gap

**Figure 5:**
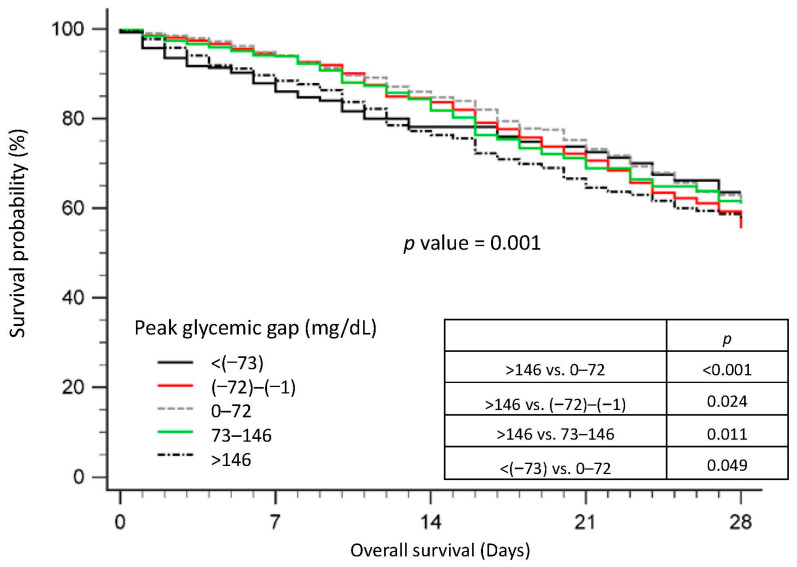
Survival curve of patients with sepsis in the intensive care unit according to Peak Glycemic gap.

Figure 5 presents the survival curves of sepsis patients categorized by their peak glycemic gap. Notably, patients with a peak glycemic gap exceeding 146 mg/dL exhibited significantly poorer survival compared to those with peak glycemic gaps of 0–72 mg/dL (*p* < 0.001), −72 to −1 mg/dL (*p* = 0.024), or 73–146 mg/dL (*p* = 0.011). Furthermore, the group with a peak glycemic gap below −73 mg/dL experienced worse survival outcomes than the group with a peak glycemic gap of 0–72 mg/dL (*p* = 0.049).

### 3.5. Prediction of Mortality in Patients with Sepsis Using Modified SOFA-pg and Modified SOFA-pgg Scores

Table 3 provides insights into the predictive capabilities of the mSOFA-pg, mSOFA-pgg, and SOFA scores on Day 3 for mortality among sepsis patients. Both the mSOFA-pg and mSOFA-pgg scores emerged as significant predictors of 28-day and 90-day mortality (*p* < 0.001).

In the subgroup of patients with mNURIC scores ≥ 6, the mSOFA-pg score demonstrated the highest predictive accuracy for mortality risk when compared to SOFA and mSOFA-pgg (28-day mortality, OR (*p*), r^2^: SOFA: 1.745 (<0.001), 0.407; mSOFA-pg: 1.689 (<0.001), 0.428; mSOFA-pgg: 1.576 (<0.001), 0.375. 90-day mortality, OR (*p*), r^2^: SOFA: 1.524 (0.001), 0.303; mSOFA-pg: 1.494 (0.001), 0.328; mSOFA-pgg: 1.359 (0.002), 0.236). In various BMI subgroups, the mSOFA-pg score exhibited strong predictive power for 90-day mortality in the underweight group (SOFA: 1.376 (<0.001), 0.255; mSOFA-pg: 1.356 (0.006), 0.325, mSOFA-pgg: 1.287 (0.014), 0.253), while mSOFA-pgg displayed good predictive power in the overweight group.

## 4. Discussion

Our findings highlight that a peak glycemic gap exceeding 146 mg/dL or falling below −73 mg/dL is associated with unfavorable survival outcomes. The utilization of the mSOFA-pgg score for predicting 7-day mortality demonstrated a substantial improvement in model fit, enhancing it by nearly 10% when compared to the conventional SOFA score. Furthermore, patients with HbA1c levels between 6.5% and 7.9% exhibited significantly improved survival rates compared to those with HbA1c levels below 6.5%. In terms of peak glucose levels, patients within the range of 141–220 mg/dL experienced prolonged survival when contrasted with those with peak glucose levels exceeding 220 mg/dL or falling below 140 mg/dL. In subgroup analyses, there were no significant differences in survival rates between patients with peak glucose levels ranging from 141–180 mg/dL and those with levels between 181–220 mg/dL. However, a distinction in mortality hazard ratios among these groups was revealed through the utilization of peak glucose levels, presenting as a U-shaped curve, with the group with peak glucose levels of 181–220 mg/dL occupying the nadir of the curve.

Our study also unveiled a noteworthy association between the disparities in peak glucose levels and average glucose levels, particularly when they exceeded 146 mg/dL or dropped below −73 mg/dL, and the increased mortality risk among sepsis patients. In alignment with prior research, fluctuations in blood glucose concentrations were found to be correlated with elevated mortality rates in sepsis patients [2,12,16,17,29]. A novel revelation from our study was that in comparison to the impact of an increase in the peak glycemic gap, a reduction in the peak glycemic gap demonstrated a lower threshold for heightened mortality risk (increase: 146 mg/dL vs. reduction: −73 mg/dL). This phenomenon may be linked to a heightened risk of hypoglycemia among patients experiencing a reduction in the peak glycemic gap. Notably, hypoglycemia is a well-established factor associated with elevated mortality rates in sepsis patients. Nevertheless, a comparative analysis of the effects of reducing in-hospital peak glycemic levels and baseline average glycemic levels has not been previously explored. Hypoglycemia is known to elevate mortality risk in the ICU, with intensive insulin therapy being a critical factor contributing to hypoglycemic events [2]. Most previous studies have relied on in-hospital average glucose levels to assess glucose variability. However, this metric does not account for chronic glycemic status and acute variability [2,18,29]. In our study, we computed the gap between peak glucose levels over a 3-day period and average glucose levels, factoring in HbA1c data from the previous three months. This approach allowed us to evaluate acute glycemic variability in relation to individual baseline chronic glycemic levels.

Building upon the findings discussed above and drawing from insights gained in our previous study, we have introduced the innovative mSOFA-pg and mSOFA-pgg scores, which hold significant predictive value for 28-day and 90-day mortality, especially within subgroups characterized by abnormal BMI or high nutritional risk.

In our prior investigation, we employed a modified SOFA score that incorporated peak glucose levels, resulting in a 5% enhancement in model fit for patients without diabetes, although the model fit remained consistent for patients with diabetes [14]. It’s important to note that a systematic review has previously reported a relatively weak or non-existent association between glycemic variability and mortality in patients with diabetes [2]. In contrast, our results bring to light a robust association between peak glucose levels, peak glycemic gap, and mortality among individuals with a history of diabetes admitted to the ICU due to sepsis. Notably, patients classified as underweight or with high mNUTRIC scores exhibited heightened sensitivity to peak glucose levels, whereas the overweight group displayed greater sensitivity to peak glycemic gaps. Consequently, the overweight group exhibited a more muted response to mSOFA-pg in comparison to mSOFA-pgg. According to a previous study, lower BMI was associated with increased glycemic variability in patients with diabetes [30]. Another study found that muscle composition was an independent predictor of the glycemic variability of patients in the ICU [31]. Lower muscle composition was associated with higher glycemic variability. Our previous study also revealed Body composition variables may be associated with sepsis outcomes [32]. Overweight, underweight, or high-risk nutrition status patients may have different body composition compared to normal weight and low-risk nutrition status patients. The interaction between different nutritional statuses and dynamic glucose change needs further study to be clarified.

The observed U-shaped hazard ratio of mortality was linked to distinct peak glucose levels. Both hyperglycemia and hypoglycemia were associated with elevated mortality risk, a pattern consistent with prior research findings [2,14,21]. In our study, the lowest mortality rate was observed among patients with peak glucose levels falling within the 181–220 mg/dL range, and their survival did not significantly differ from those with peak glucose levels > 140 mg/dL and ≤180 mg/dL. Strikingly, stringent glycemic control (typically recommended as <180 mg/dL) may potentially lead to increased mortality in critically ill patients [33,34,35]. Recent investigations have suggested that adopting a more liberal approach to glucose control may help mitigate glycemic variability and reduce the incidence of moderate-to-severe hypoglycemia [36,37]. The meta-analysis findings indicate that in comparison with intermittent glucose measurements, continuous glucose monitoring (CGM) can effectively diminish the risk of hypoglycemia, reduce overall mortality, and attenuate glycemic variability among critically ill patients [38]. Subsequent studies have also highlighted the utility of CGM in investigating the correlation between patient outcomes and a more comprehensive assessment of glycemic variability in critically ill populations. Moreover, a previous study noted that diabetic patients might tolerate higher glucose levels compared to non-diabetic individuals [19]. Our study also corroborated this finding, revealing that patients with diabetes exhibited greater tolerance for high peak glucose levels, which could be attributed to their baseline blood sugar levels being generally higher than those without diabetes. In patients with sepsis, hyperglycemia typically arises from the activation of proinflammatory mediators and the release of counterregulatory hormones, resulting in heightened peripheral insulin resistance and increased hepatic gluconeogenesis [39]. Consequently, hyperglycemia is commonly encountered in the initial presentation of sepsis patients. Furthermore, a separate study indicated that glucose levels exceeding 198 mg/dL were linked to increased mortality in patients with diabetes who maintained good glycemic control (HbA1c < 7.0%) as well as in those without diabetes. However, in that study, the peak glucose level was unable to predict mortality in patients with poor diabetes control (HbA1c ≥ 7.0%) [40]. In our study, a peak glucose level exceeding 220 mg/dL was significantly associated with heightened mortality risk among patients with diabetes. This finding suggests that the upper threshold for glycemic control targets in patients with diabetes may be relaxed to 220 mg/dL. In conclusion, patients with diabetes who can tolerate peak glucose levels ranging from 141 mg/dL to 220 mg/dL exhibited the best survival outcomes.

This study introduced a novel finding revealing that a reduction in the peak glycemic gap is associated with a lower threshold for increased mortality compared to an increase in the peak glycemic gap. Diverging from previous studies that primarily juxtaposed peak glucose levels with average glucose levels upon admission, our study introduced a variable that incorporates a comparison of patients’ baseline chronic glycemic states. As a result, this innovative design allows for a closer examination of glycemic variability in patients experiencing acute conditions. Furthermore, we devised the mSOFA-pg score, which notably improved the prediction of 28-day mortality in underweight patients with sepsis by 17.2%, and the mSOFA-pg score, which enhanced the prediction of 90-day mortality in overweight patients with sepsis by 6.8%. Another crucial finding from our study is that individuals with diabetes may exhibit peak glucose level tolerances of up to 220 mg/dL. This discovery underscores the importance of considering distinct glycemic control targets for individuals with varying underlying conditions, including those with or without diabetes and differing chronic glycemic profiles. In the future, prospective studies utilizing continuous glucose monitoring (CGM) may be undertaken in line with our glycemic control objectives to validate the advantages for patients with sepsis. Additionally, the strength of our study lies in its extensive sample size, encompassing diverse adult ICU settings (both medical and surgical) as well as various types of hospitals (referral centers and regional hospitals). This wide-ranging representation makes our findings highly applicable to a broad spectrum of patients with diabetes admitted to the ICU for sepsis.

This study is subject to certain limitations. Firstly, the retrospective study design and the exclusion of patients without HbA1c data from the preceding 3 months may introduce some selection bias. Since we lacked data on the duration of diabetes, we refrained from investigating the potential impact of diabetes duration on outcomes. It remains unclear whether patients with long-standing diabetes respond differently compared to those with recent diagnoses. Secondly, our dataset lacked specific records distinguishing between type 1 and type 2 diabetes in patients. Given their distinct disease mechanisms, these two diabetes types may exhibit varying degrees of glucose variability during the acute illness phase. However, it is worth noting that type 1 diabetes mellitus represents a very small percentage (less than 1%) of diabetes cases in Taiwan [41], which suggests a minimal impact on the present study’s outcomes. Thirdly, our observational study did not involve interventions or adjustments to glycemic control strategies before or after ICU admission [42]. Consequently, we did not implement a specific insulin treatment protocol in our study, as each patient received routine ICU blood glucose management. The variations in these protocols can help interpret the impact on peak glucose levels and glycemic variability. It is important to acknowledge that stringent blood glycemic control protocols may influence peak glucose levels. Nevertheless, a high peak glucose level, regardless of whether stringent glycemic control is applied, may indicate sepsis progression, while a low peak glucose level could hint at the occurrence of hypoglycemic episodes. These hypoglycemic events have been linked to adverse outcomes in critically ill patients [43]. However, our study lacked continuous blood glucose monitoring data, which would have allowed us to identify and analyze hypoglycemic episodes. Future prospective studies should aim to combine our models with continuous blood glucose monitoring to explore the relationship between peak glucose levels and various forms of dysglycemia, including hypoglycemia and blood glucose variability [12,44]. Furthermore, factors such as total parenteral nutrition and corticosteroid administration can influence glucose levels. Regrettably, due to limitations in the database, our study could not account for these factors. Nevertheless, in routine ICU care, stringent glycemic control is typically implemented for patients, emphasizing the significance of high peak glucose levels as an indicator of sepsis progression. Fourthly, it’s important to acknowledge potential limitations inherent in database studies, such as the presence of missing values and potential confounding factors. A prior study outlined various strategies for addressing missing data within medical databases, one of which we employed involved the removal of all variables associated with a specific sampling time if any one of them was missing [45]. While this method may result in some information loss, potentially introducing bias and reducing statistical power, it is worth noting that unlike imputing values to substitute for missing data, it does not introduce artificial or unrealistic information into the dataset. Additionally, as our study exclusively focused on ICU patients with sepsis in specific ICUs and hospitals, it is crucial to acknowledge its potential limitations in terms of the applicability of our findings to a more diverse spectrum of patient populations and varied healthcare settings. Consequently, further research is needed to extend and validate our results in different patient cohorts and healthcare contexts. Lastly, it’s important to note that the definition of sepsis according to the American Medical Association [1] underwent a change in 2016, which also led to updates to the ICD-9 and ICD-10 codes. To address this, our study employed three distinct definitions of sepsis, including the ICD-9, ICD-10, and sepsis-3 codes, to identify and include patients with sepsis. This approach aimed to minimize the loss of eligible patients [24]. Consequently, our study population aligns with the criteria of the new definition of sepsis.

## 5. Conclusions

Significantly, a peak glycemic gap exceeding 146 mg/dL or falling below −73 mg/dL, along with peak glucose levels surpassing 220 mg/dL, emerged as critical factors associated with the mortality risk among sepsis patients with diabetes. Notably, patients with diabetes demonstrated the best survival outcomes when their peak glucose levels fell within the range of 141–220 mg/dL. Furthermore, the implementation of the mSOFA-pg and mSOFA-pgg scores yielded substantial improvements in mortality prediction for sepsis patients with diabetes, particularly those with abnormal BMI or facing high nutritional risks. These findings underscore the importance of exploring precise glycemic control targets that consider diverse chronic glycemic statuses and diabetes conditions. In the future, we can consider conducting prospective studies that incorporate continuous glucose monitoring (CGM) and explore the potential influence of the duration of diabetes. These studies would align with our glycemic control goals and help confirm the benefits of glycemic control for patients with sepsis.

## Figures and Tables

**Table 1:** Baseline characteristics and outcomes of the patients with sepsis.

	HbA1c < 6.5%	6.5≤ HbA1c < 8%	HbA1c ≥ 8%	*p*	Glucose ≤ 140 (mg/dL)	140 < Glucose ≤ 220 (mg/dL)	Glucose > 220 (mg/dL)	*p*
	n = 4847	n = 6050	n = 4987		n = 998	n = 1740	n = 2647	
Age, years	67.2 (58.3–75.3)	65.5 (56.9–73.6)	60.9 (52.3–69.4)	<0.001	63.8 (54.5–73.3)	63.8 (55.5–73.2)	61.7 (53.0–70.5)	<0.001
BMI, kg/m^2^	23.3 (20.5–26.4)	24.4 (21.7–27.4)	24.6 (22.1–27.6)	<0.001	24.2 (21.6–27.4)	24.7 (22.0–27.8)	24.3 (21.8–27.2)	0.006
Gender (F), %	2088 (43.1%)	2490 (41.2%)	1999 (40.1%)	0.009	328 (32.9%)	567 (32.6%)	1068 (40.3%)	<0.001
CCI score	4.0 (2.0–6.0)	3.0 (2.0–5.0)	3.0 (2.0–5.0)	<0.001	3.0 (2.0–5.0)	3.0 (2.0–5.0)	3.0 (2.0–5.0)	0.023
DCSI	4.0 (2.0–6.0)	4.0 (2.0–6.0)	4.0 (2.0–6.0)	0.201	3.0 (2.0–5.0)	3.0 (2.0–6.0)	4.0 (2.0–6.0)	<0.001
Pneumonia	1990 (41.1%)	2338 (38.6%)	1685 (33.8%)	<0.001	289 (29.0%)	510 (29.3%)	833 (32.5%)	0.185
UTI	989 (20.4%)	1160 (19.2%)	868 (17.4%)	0.001	162 (16.2%)	230 (13.2%)	427 (16.1%)	0.019
Vasopressor	1156 (23.8%)	1369 (22.6%)	1086 (21.8%)	0.048	215 (21.5%)	266 (15.3%)	585 (22.1%)	<0.001
Ventilator	2706 (55.8%)	3288 (54.3%)	2489 (49.9%)	<0.001	420 (42.1%)	701 (40.3%)	1401 (52.9%)	<0.001
HD	1224 (25.3%)	2339 (20.3%)	856 (17.2%)	<0.001	191 (19.1%)	264 (15.2%)	467 (17.6%)	0.018
1st Day Glucose, mg/dL	159.5 (120.0–212.8)	203.0 (154.0–271.0)	299.5 (213.0–416.0)	<0.001	113.0 (92.0–127.0)	175.0 (157.0–197.0)	314.0 (256.0–418.0)	<0.001
Peak Glucose, mg/dL	163.0 (123.0–216.8)	207.0 (158.0–280.0)	307.0 (220.0–432.0)	<0.001	103.0 (93.0–128.0)	178.0 (160.0–198.0)	320.0 (262.0–432.0)	<0.001
Day1								
1st Day APACHE II	17.0 (13.0–22.0)	16.0 (12.0–21.0)	15.0 (10.0–20.0)	<0.001	15.0 (10.0–20.0)	14.0 (10.0–19.0)	16.0 (11.0–21.0)	<0.001
1st Day Temperature, °C	36.5 (36.0–37.1)	36.6 (36.1–37.2)	36.6 (36.1–37.2)	<0.001	36.6 (36.0–37.1)	36.6 (36.0–37.1)	36.6 (36.0–37.2)	0.974
1st Day SBP, mmHg	135.0 (114.0–156.0)	136.0 (115.0–155.0)	134.0 (114.0–154.0)	0.095	137.0 (113.0–157.0)	138.0 (118.0–157.0)	132.0 (113.0–154.0)	<0.001
1st Day DBP, mmHg	72.0 (60.0–84.0)	72.0 (61.0–84.0)	73.0 (62.0–86.0)	0.005	73.0 (62.0–85.0)	74.0 (63.0–86.0)	73.0 (60.0–86.0)	0.03
1st Day Pulse Pressure, mmHg	60.0 (46.0–79.0)	61.0 (46.0–77.0)	59.0 (44.0–75.0)	<0.001	60.0 (46.0–78.0)	61.0 (46.0–79.0)	58.0 (44.0–74.0)	<0.001
1st Day WBC, 1000/μL	10.4 (7.5–14.2)	10.8 (8.0–14.7)	11.1 (8.3–14.9)	<0.001	9.6 (7.2–13.1)	10.2 (7.8–13.1)	11.9 (8.7–15.8)	<0.001
1st Day qSOFA	1.0 (1.0–2.0)	1.0 (0.0–2.0)	1.0 (0.0–2.0)	0.013	1.0 (0.0–1.0)	1.0 (0.0–1.0)	1.0 (0.0–2.0)	<0.001
1st Day SOFA	5.0 (3.0–7.0)	4.0 (2.0–6.0)	4.0 (2.0–7.0)	0.010	5.0 (3.0–7.0)	4.0 (2.0–6.0)	5.0 (3.0–7.0)	<0.001
Day3	n = 4678	n = 5891	n = 4816		n = 967	n = 1714	n = 2565	
3rd Day APACHE II	15.0 (11.0–20.0)	14.0 (10.0–19.0)	13.0 (9.0–17.0)	<0.001	13.0 (9.0–18.0)	12.0 (9.0–17.0)	13.0 (9.0–18.0)	<0.001
3rd Day Temperature, °C	36.5 (36.0–37.1)	36.6 (36.1–37.1)	36.6 (36.1–37.1)	<0.001	36.5 (36.0–37.0)	36.5 (36.0–37.0)	36.6 (36.1–37.2)	0.02
3rd Day SBP, mmHg	128.0 (110.0–147.0)	128.0 (111.0–147.0)	126.0 (109.8–145.0)	<0.001	126.0 (109.0–144.0)	126.0 (110.0–146.0)	124.0 (109.0–143.0)	0.035
3rd Day DBP, mmHg	64.0 (55.0–75.0)	65.0 (55.0–75.0)	66.0 (56.0–77.0)	<0.001	65.0 (56.0–76.0)	65.0 (56.0–76.0)	64.5 (55.0–75.0)	0.122
3rd Day Pulse Pressure, mmHg	62.0 (49.0–78.0)	62.0 (49.0–78.0)	60.0 (46.0–75.0)	<0.001	59.0 (47.0–76.0)	60.0 (46.0–76.0)	60.0 (46.0–75.0)	0.472
3rd Day WBC, 1000/μL	5.9 (5.6–6.2)	7.1 (6.7–7.4)	9.3 (8.5–10.6)	<0.001	6.4 (5.8–7.2)	6.8 (6.1–7.7)	7.9 (6.9–9.7)	<0.001
3rd Day qSOFA	1.0 (1.0–2.0)	1.0 (1.0–2.0)	1.0 (1.0–2.0)	0.204	1.0 (1.0–1.0)	1.0 (0.0–1.0)	1.0 (1.0–2.0)	<0.001
3rd Day SOFA	6.0 (4.0–8.0)	5.0 (3.0–8.0)	5.0 (3.0–8.0)	<0.001	7.0 (4.0–9.0)	5.0 (4.0–8.0)	6.0 (3.0–8.0)	0.023
Mortality								
7 days	373 (7.7%)	385 (6.4%)	353 (7.1%)	0.025	64 (6.4%)	66 (3.8%)	175 (6.6%)	<0.001
28 days	917 (18.9%)	958 (15.8%)	783 (15.7%)	<0.001	165 (16.5%)	179 (10.3%)	398 (15.0%)	<0.001
90 days	1217 (26.3%)	1375 (22.7%)	1059 (21.2%)	<0.001	215 (21.5%)	246 (14.1%)	524 (19.8%)	<0.001
Hospital Mortality	1293 (26.7%)	1405 (23.2%)	1074 (21.5%)	<0.001	221 (22.1%)	250 (14.4%)	533 (20.1%)	<0.001

BMI: Body Mass Index, CCI: Charlson Comorbidity Index, DCSI: Diabetes Complications Severity Index, UTI: urinary tract infection, HD: hemodialysis. APACHE II: Acute Physiology and Chronic Health Evaluation II, SBP: systolic blood pressure, DBP: diastolic blood pressure, qSOFA: quick Sequential Organ Failure Assessment, SOFA: Sequential Organ Failure Assessment.

**Table 2:** Baseline characteristics and outcomes of the patients with sepsis who had peak glucose level >140.

Peak Glucose Level (Pg), mg/dL	140 < Pg ≤ 180 (n = 998)	180 <Pg ≤ 220 (n = 1470)	Pg > 220 (n = 2647)	*p*
Age, years	64.0 (55.6–73.4)	63.6 (55.4–72.8)	61.7 (53.0–70.5)	<0.001
BMI, kg/m^2^	24.6 (22.0–27.4)	24.9 (22.1–28.4)	24.3 (21.8–27.2)	0.002
Gender(F), %	287 (30.9%)	280 (34.5%)	1068 (40.3%)	<0.001
CCI_score	3.0 (2.0–5.0)	3.0 (2.0–5.0)	3.0 (2.0–5.0)	0.022
DCSI	3.0 (2.0–6.0)	3.0 (2.0–6.0)	4.0 (2.0–6.0)	0.014
Pneumonia	273 (29.4%)	237 (29.2%)	833 (31.5%)	0.315
UTI	112 (12.1%)	118 (14.5%)	427 (16.1%)	0.011
Vasopressor	138 (14.9%)	128 (15.8%)	586 (22.1%)	<0.001
Ventilator	351 (37.8%)	350 (43.2%)	1401 (52.9%)	<0.001
HD	143 (15.4%)	121 (14.9%)	467 (17.6%)	0.096
1st Day Glucose, mg/dL	160.0 (149.0–170.0)	199.0 (187.6–209.0)	314.0 (256.0–418.0)	<0.001
Peak Glucose, mg/dL	161.0 (151.0–170.0)	200.0 (190.0–210.0)	320.0 (262.0–432.0)	<0.001
Day 1				
1st Day APACHE II	14.0 (10.0–19.0)	14.0 (10.0–19.0)	16.0 (11.0–21.0)	<0.001
1st Day Temperature,°C	36.6 (36.0–37.1)	36.5 (36.0–37.1)	36.6 (36.0–37.2)	0.972
1st Day SBP, mmHg	139.0 (119.0–158.0)	138.0 (116.0–157.0)	132.0 (113.0–154.0)	<0.001
1st Day DBP, mmHg	75.0 (63.0–87.0)	74.0 (63.0–85.0)	73.0 (60.0–86.0)	0.016
1st Day Pulse pressure, mmHg	61.0 (48.0–78.0)	60.0 (44.8–79.0)	58.0 (44.0–74.0)	<0.001
1st Day WBC, 1000/μL	9.9 (7.6–12.6)	10.5 (8.2–13.6)	11.9 (8.7–15.8)	<0.001
1st Day qSOFA	1.0 (0.0–1.0)	1.0 (0.0–1.0)	1.0 (0.0–2.0)	<0.001
1st Day SOFA	4.0 (2.0–6.0)	4.0 (2.0–6.0)	5.0 (3.0–7.0)	<0.001
Day 3	n = 915	n = 799	n = 2565	
3rd Day APACHE II	12.0 (9.0–17.0)	13.0 (9.0–17.0)	13.0 (9.0–18.0)	<0.001
3rd Day Temperature,°C	36.6 (36.0–37.0)	36.5 (36.0–37.0)	36.6 (36.1–37.2)	0.332
3rd Day SBP, mmHg	129.0 (110.0–148.0)	124.0 (110.0–144.0)	124.0 (109.0–143.0)	0.006
3rd Day DBP, mmHg	65.0 (56.0–78.0)	65.0 (55.0–74.0)	64.5 (55.0–75.0)	0.015
3rd Day Pulse pressure, mmHg	61.0 (46.0–76.0)	60.0 (46.0–75.0)	60.0 (46.0–75.0)	0.473
3rd Day WBC, 1000/μL	6.6 (6.0–7.4)	7.0 (6.2–7.9)	7.9 (6.9–9.7)	<0.001
3rd Day qSOFA	1.0 (0.0–1.0)	1.0 (1.0–2.0)	1.0 (1.0–2.0)	<0.001
3rd Day SOFA	6.0 (4.0–9.0)	5.0 (4.0–7.0)	6.0 (3.0–8.0)	0.236
Mortality				
7 days	38 (4.1%)	28 (3.5%)	175 (6.6%)	<0.001
28 days	97 (10.4%)	82 (10.1%)	398 (15.0%)	<0.001
90 days	133 (14.3%)	113 (13.9%)	524 (19.8%)	<0.001
Hospital mortality	133 (14.3%)	117 (14.4%)	533 (20.1%)	<0.001

BMI: Body Mass Index, CCI: Charlson Comorbidity Index, DCSI: Diabetes Complications Severity Index, UTI: urinary tract infection, HD: hemodialysis. APACHE II: Acute Physiology and Chronic Health Evaluation II, SBP: systolic blood pressure, DBP: diastolic blood pressure, qSOFA: quick Sequential Organ Failure Assessment, SOFA: Sequential Organ Failure Assessment.

**Table 3:** The SOFA score day3, mSOFA-pg score, and mSOFA-pgg prediction power concerning the mortality of the participants.

	SOFA Day 3	mSOFA-pg (Peak Glucose)	mSOFA-pgg (Peak Glucose Gap)
28-day mortality OR (*p*); r^2^	1.277 (<0.001); 0.166	1.288 (<0.000); 0.209	1.286 (<0.001); 0.215
90-day mortality	1.307 (<0.001); 0.199	1.314 (<0.001); 0.245	1.301 (<0.001); 0.241
mNUTRIC score < 6			
28-day mortality OR (*p*); r^2^	1.231 (<0.001); 0.129	1.215 (<0.000); 0.129	1.204 (<0.001); 1.130
90-day mortality	1.233 (<0.001); 0.138	1.213 (<0.000);0.135	1.218 (<0.001); 0.152
mNUTRIC score ≥ 6			
28-day mortality OR (*p*); r^2^	1.745 (<0.001); 0.407	1.689 (<0.000); 0.428	1.576 (<0.001); 1.375
90-day mortality	1.524 (0.001); 0.303	1.494 (0.001); 0.328	1.359 (0.002); 0.236
BMI < 18.5			
28-day mortality OR (*p*); r^2^	1.304 (<0.001); 0.205	1.413 (0.004); 0.377	1.364 (0.007); 0.330
90-day mortality	1.376 (<0.001); 0.255	1.356 (0.006); 0.325	1.287 (0.014); 0.253
18.5 ≤ BMI < 25			
28-day mortality OR (*p*); r^2^	1.266 (<0.001); 0.148	1.242 (<0.001); 0.163	1.220 (<0.001); 0.149
90-day mortality	1.294 (<0.001); 0.180	1.288 (<0.001); 0.221	1.265 (<0.001); 0.207
BMI ≥ 25			
28-day mortality OR (*p*); r^2^	1.310 (<0.001); 0201	1.347 (<0.001); 0.258	1.398 (<0.001); 0.310
90-day mortality	1.343 (<0.001); 0.237	1.365 (<0.001); 0.283	1.378 (<0.001); 0.305

OR: odds ratio; mNUTRIC score: modified Nutrition Risk in Critically ill score; BMI: Body Mass Index.

## Data Availability

The datasets used and analyzed in the current study are available from the corresponding author upon reasonable request.

## Supplementary Materials

The following supporting information can be downloaded at: https://www.mdpi.com/article/10.3390/nu15183973/s1, Table S1. Modified NUTRIC score. Table S2. Baseline characteristics and outcomes of the patients with sepsis. Table S3. Baseline characteristics and outcomes of the patients with sepsis who had peak glucose level ≤ 140. Figure S1. Flowchart of patient selection. Figure S2. HbA1C and in-hospital mortality of patients with sepsis in intensive care unit.

## Abbreviations

HbA1c: glycated hemoglobin; DM: diabetes mellitus; ICU: intensive care unit; DCSI: Diabetes Complications Severity Index; SOFA: Sequential Organ Failure Assessment; mNUTRIC (modified Nutrition Risk in the Critically) score; BMI: Body Mass Index; ICD-9: The International Statistical Classification of Diseases and Related Health Problems, 9th Revision; ICD-10: The International Statistical Classification of Diseases and Related Health Problems, 10th Revision; CI: confidence interval; HR: hazard ratio; CCI: Charlson comorbidity index; SD: standard deviation; IQR: Interquartile range; CV: cardiovascular; CNS: central nervous system; KM: Kaplan–Meier; WBC: blood leukocytes; qSOFA: quick SOFA; BP: blood pressure; mSOFA-pg: Modified Sequential Organ Failure Assessment-Peak Glucose Score mSOFA-pgg: Modified Sequential Organ Failure Assessment–Peak Glycemic Gap.
