# Supplementary material for: Use of Peak Glucose Level and Peak Glycemic Gap in Mortality Risk Stratification in Critically Ill Patients with Sepsis and Prior Diabetes Mellitus of Different Body Mass Indexes
Source: Nutrients. 2023 Sep 14;15(18):3973. doi: 10.3390/nu15183973 (PMC10534504; doi:10.3390/nu15183973)
Supplement: Supplementary file 1 [file nutrients-15-03973-s001.zip › nutrients-2601792-supplementary.pdf]

Table S1. modified NUTRIC score.

Variable	Range	Points
Age	<50	0
	≥50 and <75	1
	≥75	2
APACHE II	<15	0
	≥15 and <20	1
	≥20 and <28	2
	≥28	3
SOFA	<6	0
	≥6 and <10	1
	≥10	2
Number of comorbidities	0–1	0
	≥2	1
Days from hospital to ICU admission	≥0 and <1	0
	≥1	1

Table S2 Baseline characteristics and outcomes of the patients with sepsis

	HbA1C≤7	HbA1C>7	*p*	Pg ≤140	140< Pg ≤220	Pg >220	*p*
Patients number	n=7799	n=8085		n=998	n=1740	n=2647	
Age	66.8 (58.2-74.9)	62.3 (53.6-70.9)	<0.001	63.8 (54.5-73.3)	63.8 (55.5-73.2)	61.7 (53.0-70.5)	<0.001
BMI	23.7 (20.9-26.9)	24.6 (22.0-27.5)	<0.001	24.2 (21.6-27.4)	24.7 (22.0-27.8)	24.3 (21.8-27.2)	0.006
Gender(F)	3322 (42.6%)	3255(40.3%)	0.003	328(32.9%)	567(32.6%)	1068(40.3%)	<0.001
CCI score	3.0 (2.0-6.0)	3.0 (2.0-5.0)	<0.001	3.0 (2.0-5.0)	3.0 (2.0-5.0)	3.0 (2.0-5.0)	0.023
DCSI	4.0 (2.0-6.0)	4.0 (2.0-6.0)	0.27	3.0 (2.0-5.0)	3.0 (2.0-6.0)	4.0 (2.0-6.0)	<0.001
Pneumonia	4127(40.1%)	2886(35.7%)	<0.001	289(29.0%)	510(29.3%)	833(32.5%)	0.185
UTI	1581(20.3%)	1436(17.8%)	<0.001	162(16.2%)	230(13.2%)	427(16.1%)	0.019
Vasopressor	1811(23.2%)	1800(22.3%)	0.15	215(21.5%)	266(15.3%)	585(22.1%)	<0.001
Ventilator	4290(55%)	4193(51.9%)	<0.001	420(42.1%)	701(40.3%)	1401(52.9%)	<0.001
HD	1831(23.5%)	1478(18.3%)	<0.001	191(19.1%)	264(15.2%)	467(17.6%)	0.018
1st Day Glucose	169.0 (129.0-229.0)	265.0 (188.0-377.0)	<0.001	113.0 (92.0-127.0)	175.0 (157.0-197.0)	314.0 (256.0-418.0)	<0.001
Peak Glucose	172.0 (133.0-232.0)	271.0 (194.0-388.0)	<0.001	103.0 (93.0-128.0)	178.0(160.0-198.0)	320.0(262.0-432.0)	<0.001
Day1							
1st Day APACHE II	17.0 (12.0-22.0)	15.0 (11.0-20.0)	<0.001	15.0 (10.0-20.0)	14.0 (10.0-19.0)	16.0 (11.0-21.0)	<0.001
1st Day Temperature	36.6 (36.0-37.1)	36.6 (36.1-37.2)	<0.001	36.6 (36.0-37.1)	36.6 (36.0-37.1)	36.6 (36.0-37.2)	0.974
1st Day SBP	135.0 (114.0-156.0)	134.5 (115.0-155.0)	0.399	137.0 (113.0-157.0)	138.0 (118.0-157.0)	132.0 (113.0-154.0)	<0.001
1st Day DBP	72.0 (60.0-84.0)	73.0 (62.0-85.0)	0.005	73.0 (62.0-85.0)	74.0 (63.0-86.0)	73.0 (60.0-86.0)	0.03
1st Day Pulse pressure	61.0 (46.0-78.0)	60.0 (45.0-76.0)	0.002	60.0 (46.0-78.0)	61.0 (46.0-79.0)	58.0 (44.0-74.0)	<0.001

1st Day WBC	10.5 (7.6-14.4)	11.1 (8.3-14.9)	<0.001	9.6 (7.2-13.1)	10.2 (7.8-13.1)	11.9 (8.7-15.8)	<0.001
1st Day qSOFA	1.0 (0.0-2.0)	1.0 (0.0-2.0)	0.03	1.0 (0.0-1.0)	1.0 (0.0-1.0)	1.0 (0.0-2.0)	<0.001
1st Day SOFA	5.0 (2.0-7.0)	4.0 (2.0-7.0)	0.786	5.0 (3.0-7.0)	4.0 (2.0-6.0)	5.0 (3.0-7.0)	<0.001
Day3	n=7552	n=7833		n=967	N=1714	N=2565	
3rd Day APACHE II	15.0 (11.0-19.0)	13.0 (9.0-18.0)	<0.001	13.0 (9.0-18.0)	12.0 (9.0-17.0)	13.0 (9.0-18.0)	<0.001
3rd Day Temperature	36.5 (36.0-37.1)	36.6 (36.1-37.1)	0.006	36.5 (36.0-37.0)	36.5 (36.0-37.0)	36.6 (36.1-37.2)	0.02
3rd Day SBP	128.0 (111.0-147.0)	126.0 (110.0-145.0)	0.026	126.0 (109.0-144.0)	126.0 (110.0-146.0)	124.0 (109.0-143.0)	0.035
3rd Day DBP	64.0 (55.0-75.0)	65.0 (56.0-76.0)	<0.001	65.0 (56.0-76.0)	65.0 (56.0-76.0)	64.5 (55.0-75.0)	0.122
3rd Day Pulse pressure	62.0 (49.0-78.0)	60.0 (47.0-76.0)	<0.001	59.0 (47.0-76.0)	60.0 (46.0-76.0)	60.0 (46.0-75.0)	0.472
3rd Day WBC	6.3 (5.8-6.6)	8.3 (7.5-9.7)	<0.001	6.4 (5.8-7.2)	6.8 (6.1-7.7)	7.9 (6.9-9.7)	<0.001
3rd Day qSOFA	1.0 (1.0-2.0)	1.0 (1.0-2.0)	0.446	1.0 (1.0-1.0)	1.0 (0.0-1.0)	1.0 (1.0-2.0)	<0.001
3rd Day SOFA	6.0 (4.0-8.0)	5.0 (3.0-8.0)	0.022	7.0 (4.0-9.0)	5.0 (4.0-8.0)	6.0 (3.0-8.0)	0.023
Mortality							
7-days	548(7%)	543(7%)	0.876	64(6.4%)	66(3.8%)	175(6.6%)	<0.001
28-days	1346(17.3%)	1312(16.2%)	0.082	165(16.5%)	179(10.3%)	398(15.0%)	<0.001
90-days	1916(24.6%)	1789(22.1%)	<0.001	215(21.5%)	246(14.1%)	524(19.8%)	<0.001
Hospital mortality	1953(25.0%)	1819(22.5%)	<0.001	221(22.1%)	250(14.4%)	533(20.1%)	<0.001

Pg: peak glucose level

Table S3 Baseline characteristics and outcomes of the patients with sepsis who had peak glucose level ≤140

Peak glucose level (Pg)	Pg ≤ 80 (n=145)	80 < Pg ≤ 110 (n=308)	110 < Pg ≤ 140 (n=545)	*p*
Age	63.0(55.2-74.1)	63.4(55.0-72.7)	64.0(54.1-73.5)	0.920
BMI	23.4(20.4-26.8)	24.0(21.7-27.4)	24.7(21.8-27.4)	0.021
Gender(F)	42(29.0%)	106(34.4%)	180(33.0%)	0.511
CCI_score	4.0(2.0-6.0)	3.0(2.0-5.0)	3.0(2.0-5.0)	<0.001
DCSI	3.0(2.0-5.0)	3.0(2.0-5.0)	3.0(2.0-6.0)	0.082
Pneumonia	65(44.8%)	84(27.3%)	140(25.7%)	<0.001
UTI	28(19.3%)	58(18.8%)	76(13.9%)	0.098
Vasopressor	52(35.9%)	63(20.5%)	100(18.3%)	<0.001
Ventilator	90(62.1%)	127(41.2%)	203(37.2%)	<0.001
HD	38(26.2%)	62(20.1%)	91(16.7%)	0.031
1st Day Glucose	58.0(42.0-73.0)	98.0(90.0-105.0)	126.0(118.0-134.0)	<0.001
Peak Glucose	58.0(42.0-73.0)	98.5(90.0-105.0)	127.0(119.0-134.0)	<0.001
Day1				
1st Day APACHE II	19.0(14.0-24.0)	15.0(10.0-20.0)	14.0(9.0-19.0)	<0.001
1st Day Temperature	36.7(36.1-37.3)	36.5(36.0-37.1)	36.6(36.1-37.1)	0.092
1st Day SBP	131.5(108.8-158.0)	138.0(112.8-158.0)	138.0(115.0-156.0)	0.622
1st Day DBP	70.5(58.8-82.3)	73.0(62.0-87.0)	74.0(63.0-84.3)	0.430
1st Day Pulse pressure	58.0(43.8-79.3)	60.0(47.0-77.3)	60.0(46.0-78.0)	0.847
1st Day WBC	10.7(7.3-15.2)	9.0(7.0-12.9)	9.6(7.3-12.5)	0.058
1st Day qSOFA	1.0(1.0-2.0)	1.0(0.0-1.0)	1.0(0.0-1.0)	<0.001
1st Day SOFA	6.5(4.0-9.0)	5.0(3.0-8.0)	4.0(1.5-7.0)	<0.001
Day3	n=133	n=300	n=534	
3rd Day APACHE II	17.0(12.0-22.0)	12.0(9.0-17.0)	12.0(8.5-17.0)	<0.001

3rd Day Temperature	36.5(36.1-37.1)	36.4(36.0-37.0)	36.5(36.0-37.0)	0.165
3rd Day SBP	129.0(111.8-149.0)	129.0(110.0-146.0)	123.0(108.0-142.0)	0.182
3rd Day DBP	62.0(52.8-77.5)	65.0(55.0-77.0)	65.0(56.0-75.0)	0.606
3rd Day Pulse pressure	64.0(48.0-79.0)	60.0(48.0-76.0)	58.0(45.0-74.8)	0.081
3rd Day WBC	6.4(5.8-7.1)	6.3(5.9-7.3)	6.4(5.8-7.3)	0.569
3rd Day qSOFA	1.0(1.0-2.0)	1.0(0.0-1.0)	1.0(0.0-1.0)	0.006
3rd Day SOFA	8.0(4.5-11.0)	7.0(4.0-10.0)	6.0(3.0-9.0)	0.092
Mortality				
7-days	22(15.2%)	18(5.8%)	24(4.4%)	<0.001
28-days	44(30.5%)	52(16.9%)	69(12.7%)	<0.001
90-days	63(43.4%)	63(20.5%)	89(16.3%)	<0.001
Hospital mortality	66(45.5%)	64(20.8%)	91(16.7%)	<0.001

BMI: Body Mass Index, CCI: Charlson Comorbidity Index, DSCI: Diabetes Complications Severity Index, UTI: Urinary tract infection, HD: Hemodialysis. APACHE II :Acute Physiology and Chronic Health Evaluation II, SBP: Systolic blood pressure, DBP: Diastolic blood pressure, qSOFA: quick Sequential Organ Failure Assessment, SOFA: Sequential Organ Failure Assessment

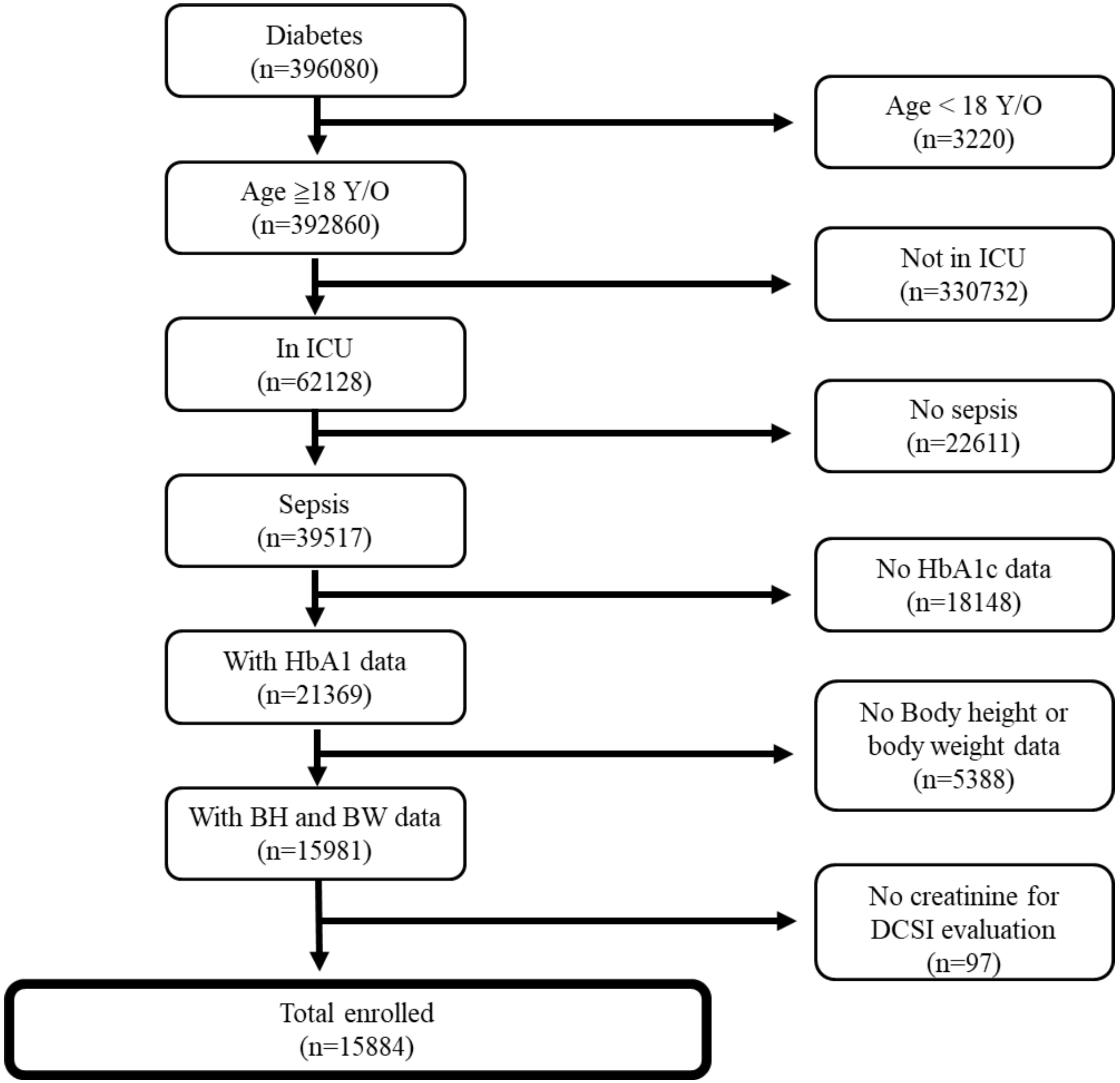

Figure S1. Flowchart of patient selection

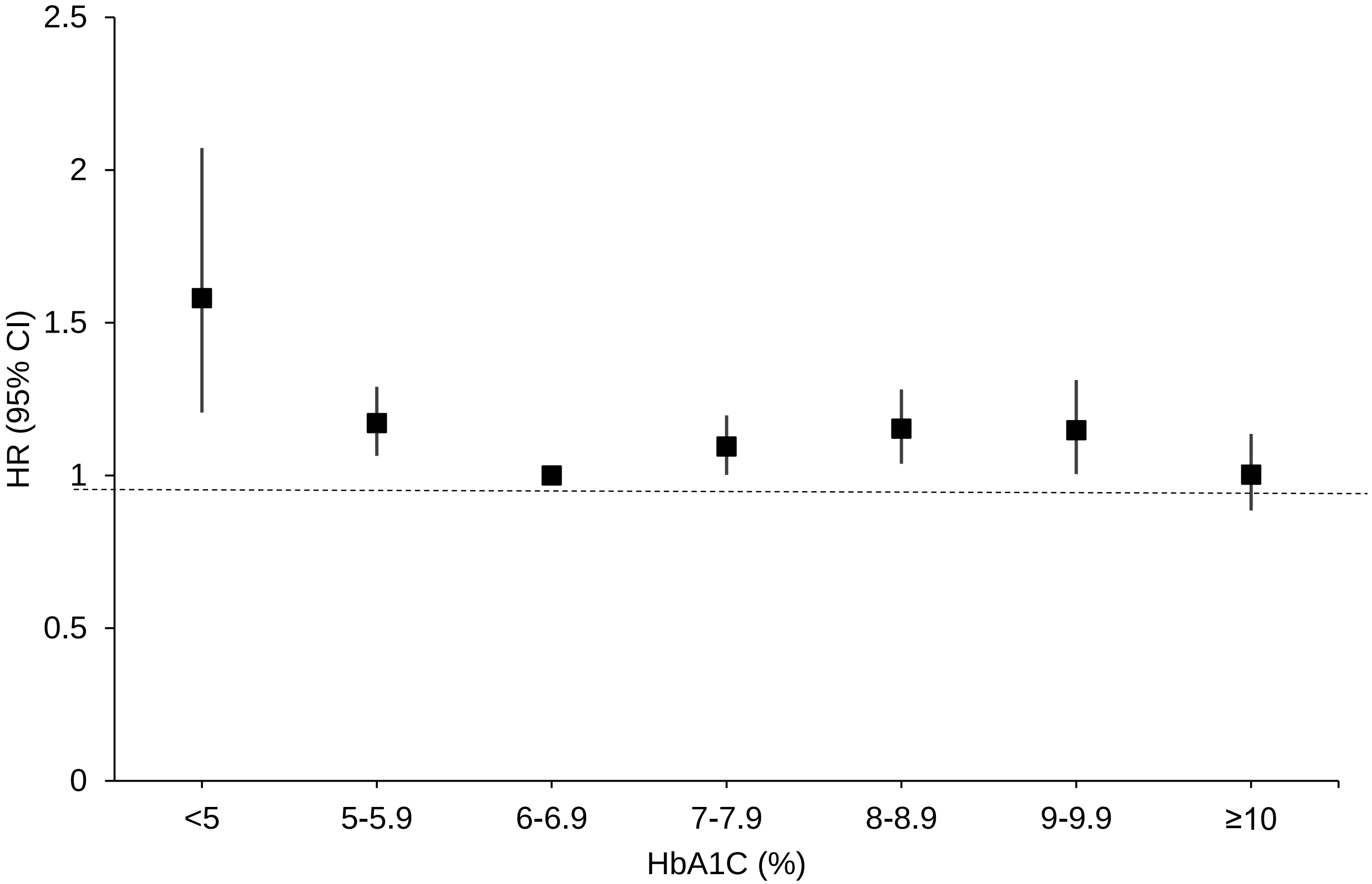

Figure S2. HbA1C and in-hospital mortality of patients with sepsis in intensive care unit
